# Spontaneous early-onset neurodegeneration in the brainstem and spinal cord of NSG, NOG, and NXG mice

**DOI:** 10.1177/03009858231151403

**Published:** 2023-02-02

**Authors:** Giovanni Finesso, Elinor Willis, James Carmine Tarrant, Matthew Lanza, Justin Sprengers, Jillian Verrelle, Esha Banerjee, Els Hermans, Charles-Antoine Assenmacher, Enrico Radaelli

**Affiliations:** 1 Comparative Pathology Core, Department of Pathobiology, School of Veterinary Medicine, University of Pennsylvania, Philadelphia, PA, USA; 2 GlaxoSmithKline, Collegeville, PA, USA; 3 Penn State University, Hershey, PA, USA; 4 Netherlands Cancer Institute, Amsterdam, The Netherlands

**Keywords:** CD34, gliosis, neurodegeneration, NOG, NSG, NXG, spontaneous lesion, vacuolation

## Abstract

The spectrum of background, incidental, and experimentally induced lesions affecting NSG and NOG mice has been the subject of intense investigation. However, comprehensive studies focusing on the spontaneous neuropathological changes of these immunocompromised strains are lacking. This work describes the development of spontaneous early-onset neurodegeneration affecting both juvenile and adult NSG, NOG, and NXG mice. The study cohort consisted of 367 NSG mice of both sexes (including 33 NSG-SGM3), 61 NOG females (including 31 NOG-EXL), and 4 NXG females. These animals were primarily used for preclinical CAR T-cell testing, generation of humanized immune system chimeras, and/or tumor xenograft transplantation. Histopathology of brain and spinal cord and immunohistochemistry (IHC) for AIF-1, GFAP, CD34, and CD45 were performed. Neurodegenerative changes were observed in 57.6% of the examined mice (affected mice age range was 6-36 weeks). The lesions were characterized by foci of vacuolation with neuronal degeneration/death and gliosis distributed throughout the brainstem and spinal cord. IHC confirmed the development of gliosis, overexpression of CD34, and a neuroinflammatory component comprised of CD45-positive monocyte-derived macrophages. Lesions were significantly more frequent and severe in NOG mice. NSG males were considerably more affected than NSG females. Increased lesion frequency and severity in older animals were also identified. These findings suggest that NSG, NOG, and NXG mice are predisposed to the early development of identical neurodegenerative changes. While the cause of these lesions is currently unclear, potential associations with the genetic mutations shared by NSG, NOG, and NXG mice as well as unidentified viral infections are considered.

NOD. Cg-*Prkdc*^scid^*Il2rg*^tm1Wjl^/SzJ (NSG), NOD. Cg-*Prkdc*^scid^*Il2rg*^tm1Sug^/JicTac (NOG), and NOD-*Prkdc*^scid^*Il2rg*^Tm1^/Rj (NXG) mice carry loss-of-function mutations of the *Prkdc* and *Il2rg* genes onto a NOD (nonobese diabetic) background.^8,29^ Because of their severely immunodeficient status, these mouse lines are extensively used in the preclinical setting as recipients for human tumor, immune cell, and stem cell transplantation.^12,25^ Even though the 3 mouse strains feature distinct *Il2rg* targeted mutations and different NOD backgrounds, these models are considered equivalent in terms of the overall biological/physiological characteristics and experimental applications.^22^

The study of naturally occurring and experimentally induced lesions affecting NSG and NOG has been the subject of intense investigation by veterinary pathologists.^2,29,34,40,41^ Notably, the increased prevalence and severity of inclusion body nephropathy in NSG mice have led to the discovery of the novel mouse kidney parvovirus.^31^ Furthermore, a series of independent studies have elucidated the pathogenesis of unintended post-transplant disorders in NSG or NOG mice engrafted with patient-derived xenografts (PDX) or human hematopoietic stem cells (HSC).^2,11,29,38,41^ Collectively, these works have exposed significant limitations in the experimental utilization of humanized NSG (including next-generation models such as NSG-SGM3) and NOG mice.^1,11,38^ Compared with the NSG and NOG, little is known about the NXG mouse line, which was generated and commercialized by Janvier laboratories in 2020 (https://janvier-labs.com/en/fiche_produit/1-nxg-immunodeficient-mouse/). Nevertheless, it is assumed that this latter model suffers from similar spontaneous and experimentally induced conditions.

Comprehensive studies focusing on spontaneous neuropathological changes affecting NSG, NOG, and NXG mice are lacking. Despite neurodegenerative lesions being infrequently reported, there are sporadic descriptions of distinct “spongiotic” changes involving the brainstem and spinal cord of both NSG and NOG mouse strains.^14,22^ Interestingly, in our collective experience as mouse pathologists for the past 10 years, we have gathered ample evidence of comparable lesions affecting the central nervous system (CNS) of juvenile and adult NSG, NOG, and NXG mice from different studies conducted in multiple facilities across Europe and the United States.

Therefore, the scope of this work is to provide a comprehensive description of the nature and frequency of this distinct neuropathological phenotype in a large-scale observational study that includes the systematic assessment of the brain and spinal cord in 432 mice with NSG, NOG, or NXG genetic backgrounds. Our investigation confirms that all of these mouse lines are prone to developing spontaneous early-onset neurodegeneration in the brainstem and spinal cord. While the exact cause of these lesions is currently unclear, we speculate on the hypothetical role of the epistatic interaction between the mutations shared by NSG, NOG, and NXG mice. The possible implication of unidentified viral infections is also discussed.

## Materials and Methods

### Animals

This observational study encompassed the neuropathological assessment of 432 mice with an NSG, NOG, or NXG genetic background. Data were extrapolated from 33 studies conducted in different research facilities across Europe and the United States between 2013 and 2022. The mice were primarily used for preclinical CAR T-cell testing, generation of chimeric animals with humanized immune system, and/or tumor xenograft transplantation. Neuropathological data of 50 C57BL/6J mice of a comparable age range and used in similar experimental settings (ie, CAR T-cell testing and tumor transplantation experiments) were also included to demonstrate the significant association between the described phenotype and the NSG, NOG, or NXG mouse cohorts. For all animals in the study, complete necropsies with macroscopic postmortem examinations and full histopathological assessments (including neuropathology) were performed as previously described.^38^ Based on previous studies,^13,39^ the following age categories were identified within the study population: juvenile (9-week-old or younger), young adult (10 to 26 weeks of age), and adult (27 to 77 weeks of age). Demographic data concerning the mouse groups included in this work are summarized in Table 1. For each individual mouse considered in this study, a comprehensive summary of animal and experimental data is provided in Supplemental Table S1.

**Table 1.** Demographic data concerning the mouse groups included in the study.

Mouse strain	All NSG, NOG, and NXG^a^	All NSG^b^	All NOG^c^	NSG	NSG-SGM3	NOG	NOG-EXL	NXG	C57BL/6
Total number of mice	432	367	61	334	33	30	31	4	50
Number of males	155	155	0	137	18	0	0	0	4
Number of females	277	212	61	197	15	30	31	4	46
Average age (weeks)	20.1	19.3	24.7	18.9	24.2	24.9	24.5	16	13.1
Median age (weeks)	22	20	28	20	24	28	28	16	13
Age range (weeks)	31 (6–37)	31 (6–37)	25 (11–36)	31 (6–37)	7 (19–26)	21 (11–32)	24 (12–36)	0	23 (7–30)
Interquartile age range (weeks)	11 (14–25)	10 (14–24)	16 (16–32)	10 (14–24)	3 (23–26)	13 (19–32)	16 (16–32)	0	9 (7–16)
Number of juveniles	58	58	0	58	0	0	0	0	15
Number of young adults	326	296	26	263	33	13	13	4	32
Number of adults	48	13	35	13	0	17	18	0	3
Number of experimentally naive mice	59	43	12	33	10	7	5	4	3
Number of experimentally manipulated mice	373	324	49	301	23	23	26	0	47

a Comprises all mice on NSG (ie, NSG and NSG-SGM3), NOG (ie, NOG and NOG-EXL), and NXG backgrounds.

b Comprises all mice on an NSG background (ie, NSG and NSG-SGM3).

c Comprises all mice on a NOG background (ie, NOG and NOG-EXL).

Mice were kept under similar husbandry conditions within specific pathogen-free facilities. All animal experiments were performed following the Institutional Animal Care and Use Committee guidelines of the mouse facilities involved in the study including the University of Pennsylvania (IACUC protocol #806175), KU Leuven (IACUC protocol #072/2015), Penn State Hershey Medical Center (IACUC protocol #PRAMS200746915), and the Netherlands Cancer Institute (IACUC protocol #11.25.8624).

### Brain and Spinal Cord Collection and Pathological Examination

All animals included in this study were euthanized via carbon dioxide asphyxiation. In most cases, the whole head and spine were collected during necropsy and immersion fixed in 10% neutral buffered formalin (NBF) for at least 10 days. To allow more rapid penetration of the fixative solution into the cranial cavity, 4 holes were drilled with a 26-gauge needle along the midsagittal parietal and frontal sutures of the skull

Upon complete fixation, most of the heads and all spines were decalcified in a solution of 15% formic acid for 24 hours. Decalcified heads were coronally sliced using the following anatomical landmarks: base of the ear canals (caudal profile, which includes medulla oblongata and cerebellum), base of the ear canals (rostral profile, which includes pons, midbrain, caudal hippocampus, and occipital cortical regions), midway between ear canals and orbits (which includes hypothalamus, thalamus, rostral hippocampus, and temporoparietal cortical regions), caudal orbital edge (which includes the base of the olfactory bulbs and frontal cortex), rostral orbital edge, and midway between the eyes and the tip of the nose (no brain regions represented in these 2 latter planes of section). Cross sections of decalcified spinal segments from the cervical, thoracic, and lumbar regions were also obtained from each animal. For some studies, the brains were carefully removed from the skull after fixation and trimmed, using a mouse brain matrix (BSMYS001-1; Zivic Instruments, Pittsburgh, PA). Five coronal slices were then obtained using the following anatomical landmarks: caudal profile of paraflocculi (which includes medulla oblongata, and cerebellum), caudal edge of mammillary bodies (which includes pons, midbrain, caudal hippocampus, and occipital cortical regions), caudal profile of the optic chiasm (which includes hypothalamus, thalamus, rostral hippocampus, and temporoparietal cortical regions), the most ventral aspect of the olfactory tubercles (which includes olfactory tubercles, basal ganglia, and frontal cortex), and base of the olfactory bulbs (which includes olfactory bulbs and frontal cortex). All samples were routinely processed for paraffin embedding, sectioned at 5 µm, and stained with hematoxylin and eosin (HE).

IHC for AIF-1, GFAP, CD34, human-specific CD45 leukocyte common antigen (LCA), and mouse-specific CD45 LCA was performed on additional sections obtained from selected cases. Multiplex immunofluorescence (IF) was also utilized to study the colocalization between CD34 and AIF-1 or GFAP. The materials and methods concerning IHC and IF, as well as the number of cases considered for each marker, are detailed in Supplemental Table S2. Fluoro-Jade C histochemistry was performed on a selected subset of samples (ie, brains from 5 affected NSG, 2 normal NSG, and 2 normal C57BL/6J mice) as previously described.^35,39^

The histopathological evaluation was conducted by 4 board-certified veterinary pathologists (ie, JCT, ML, CAA, and ER) with specific rodent pathology and neuropathology expertise.

### Statistical Analysis

Statistical analyses were performed using the GraphPad Prism 9.4 software. Chi-square for trend and Fisher’s exact tests were used to analyze variations in the frequency and severity of neurodegenerative lesions across the different mouse strain, age, sex, and/or experimental groups identified in the study population. Average age comparisons were performed using the Student’s *t* test. *P* < .05 was considered statistically significant. NXG mice were not included in the statistical analyses comparing the different mouse lines because of the small group size.

## Results

Distinct neurodegenerative changes were reported in 57.6% of the examined NSG, NOG, and NXG mice considered in this study with a prevalence of 53.1% in the NSG group, 82% in the NOG group, and 100% in the NXG group. Neurodegeneration was not observed in any of the C57BL/6J mice included as controls. The frequency, severity, and distribution of these changes across different mouse strain, age, and/or sex groups identified in the study population are summarized in Table 2. A comprehensive summary of demographic, experimental, and neuropathological data for each individual mouse considered in this study is provided in Supplemental Table 1.

**Table 2.** Overview of the mouse cohorts included in the study summarizing the frequency and severity of neurodegenerative changes based on the different sex groups, age categories, experimental use, and anatomical distribution.

Mouse strain	All NSG, NOG, and NXG^a^	NSG^b^	NOG^c^	NXG	C57BL/6
Total affected/total examined (%)	249/432 (57.6%)	195/367 (53.1%)	50/61 (82%)	4/4 (100%)	0/50 (0%)
Affected males/total males (%)	100/155 (64.5%)	100/155 (64.5%)	na	na	0/4 (0%)
Affected females/total females (%)	149/277 (53.8%)	95/212 (45%)	50/61 (82%)	4/4 (100%)	0/46 (0%)
Affected juveniles/total juveniles (%)	27/58 (46.6%)	27/58 (46.6%)	na	na	0/15 (0%)
Affected young adults/total young adults (%)	180/326 (55.2%)	159/296 (53.7%)	17/26 (65.3%)	4/4 (100%)	0/32 (0%)
Affected adults/total adults (%)	42/48 (87.5%)	9/13 (69.2%)	33/35 (94.3%)	na	0/3 (0%)
Cases with both brain and spinal cord involvement/total affected (%)	130/249 (52.2%)	93/195 (47.7%)	34/50 (68%)	3/4 (75%)	na
Cases with only brain involvement/total affected (%)	105/249 (42.2%)	90/195 (46.2%)	14/50 (28%)	1/4 (25%)	na
Cases with only spinal cord involvement/total affected (%)	14/249 (5.6%)	12/195 (6.2%)	2/50 (4%)	0/4 (0%)	na
Severe-to-moderate cases/total affected (%)	47/249 (18.9%)	32/195 (16.4%)	15/50 (30%)	0/4 (0%)	na
Severe-to-moderate cases in males/affected males (%)	18/100 (18%)	18/100 (18%)	na	na	na
Severe-to-moderate cases in females/affected females (%)	29/149 (19.5%)	14/95 (15%)	15/50 (30%)	0/4 (0%)	na
Severe-to-moderate cases in juveniles/Affected juveniles (%)	2/27 (7.4%)	2/27 (7.4%)	na	na	na
Severe-to-moderate cases in young adults/affected young adults (%)	28/180 (15.6%)	24/159 (15.1%)	4/17 (23.5%)	0/4 (0%)	na
Severe-to-moderate cases in adults/affected adults (%)	17/42 (40%)	6/9 (66.7%)	11/33 (33.3%)	na	na
Cases in experimentally naive mice/experimentally naive mice (%)	48/59 (81.4%)	34/43 (79.1%)	10/12 (83.3%)	4/4 (100%)	0/3 (0%)
Cases in experimentally manipulated mice/experimentally manipulated mice (%)	201/373 (53.9%)	161/324 (49.7%)	40/49 (81.6%)	na	0/47 (0%)

Abbreviation: na, not applicable.

a Comprises all mice on NSG (ie, NSG and NSG-SGM3), NOG (ie, NOG and NOG-EXL), and NXG backgrounds.

b Comprises all mice on an NSG background (ie, NSG and NSG-SGM3).

c Comprises all mice on a NOG background (ie, NOG and NOG-EXL).

**Figure 1.**
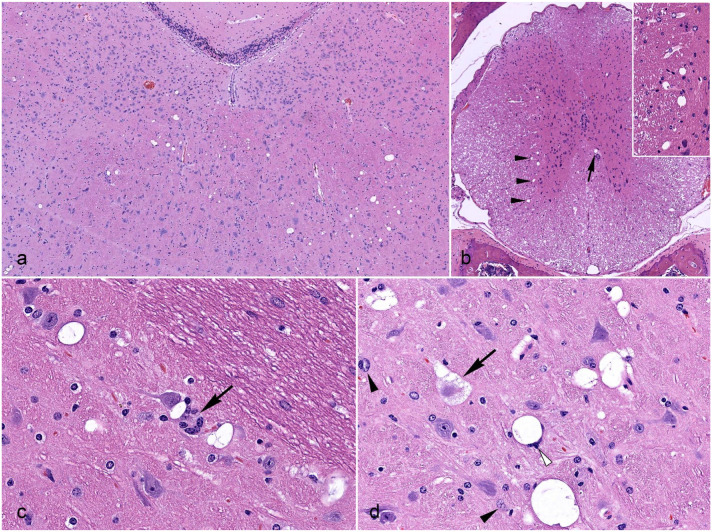
Histopathologic features of spontaneous neurodegeneration in NSG and NOG mice. Hematoxylin and eosin. (a) Pons from an 11-week-old NSG female. A locally extensive region of the reticular formation shows mild-to-moderate vacuolation of the neuroparenchyma. (b) Thoracic spinal cord from an 11-week-old NSG female. Minimal vacuolation is evident along the gray/white matter junction of ventral horns (arrowheads and inset) and intermediate gray (arrow). (c) Medulla oblongata from a 17-week-old NOG female. Mild neurodegenerative changes are characterized by small individual vacuoles accompanied by aggregates of microglial cells (arrow).(d) Medulla oblongata from a 25-week-old NOG female. Moderate neurodegenerative changes are characterized by clusters of large vacuoles often containing wispy debris. Neuroparenchymal vacuolation is accompanied by gliosis with scattered reactive astrocytes (black arrowheads), and microglial cells clustered around a vacuole (white arrowhead). A degenerating/dying neuron with cytoplasmic vacuolation and nuclear fading (“ghost cell”) is also evident (arrow).

Histologically, the lesions were all characterized by foci of neuroparenchymal vacuolation associated with gliosis and neuronal degeneration/death. These changes were multifocally distributed throughout the brainstem (especially in the reticular formation of pons and medulla oblongata) (Fig. 1a) and spinal cord (mainly affecting the gray/white matter junction of ventral horns and intermediate gray matter) (Fig. 1b). While in most instances (52.2%) both the brain and spinal cord were concurrently affected, a number of cases (42.2%) were characterized by brain involvement without spinal cord lesions. Conversely, mice with spinal cord lesions without brain involvement were rare (5.6%).

In most cases (81.1%), the neurodegenerative changes were graded as minimal or mild. In these lesions, scattered neuroparenchymal vacuoles (ranging from 5 to 20 µm in diameter) and minimal gliosis were the only detectable findings (Fig. 1c). Moderate or severe lesions were rare (18.9%) and almost exclusively observed at the level of pons and medulla oblongata, whereas changes in the spinal cord tended to be minimal or mild. Neurodegenerative changes graded as moderate or severe were characterized by focally extensive neuroparenchymal vacuolation with prominent gliosis and evidence of individual neuronal degeneration and death. More severe lesions featured clusters of larger vacuoles (up to 50 µm in diameter) often containing amphophilic debris (Fig. 1d). Degenerating/dying neurons appeared swollen with variable degrees of vacuolation and/or fading including pale cytoplasm and indistinct nuclei (“ghost cells”) (Fig. 1d and Supplemental Figure S1). Swelling and vacuolation of glial cells were occasionally observed in association with neuronal degeneration and death. Fluoro-Jade C-positive neurons were not identified.

**Figure 2.**
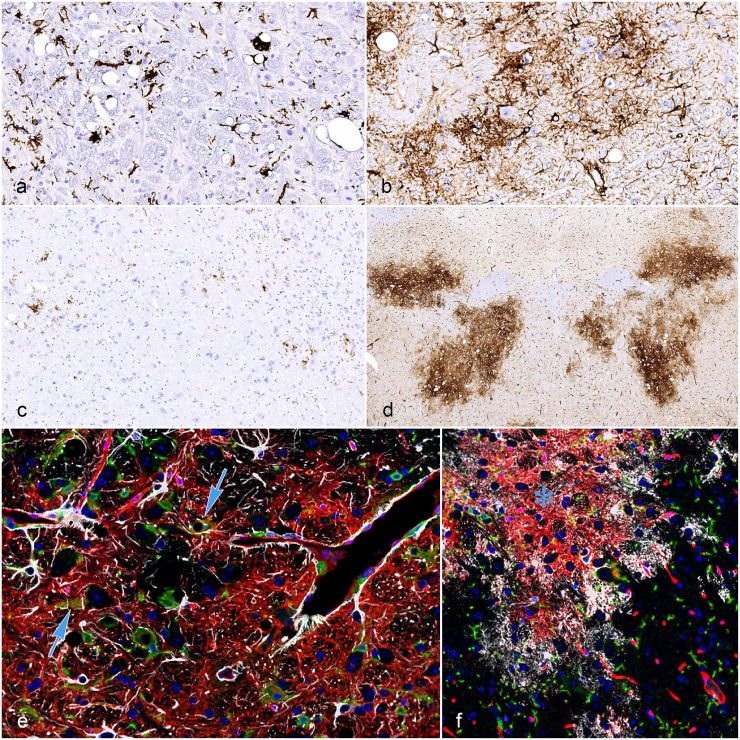
Immunohistochemical features of spontaneous neurodegeneration in NSG and NOG mice. (a) Medulla oblongata from a 25-week-old NSG male. AIF-1 immunohistochemistry (IHC) demonstrates the prominent microgliosis associated with the neurodegenerative lesions including microglial cells clustered around individual vacuoles. DAB immunoperoxidase labeling with hematoxylin counterstain. (b) Medulla oblongata from a 25-week-old NSG male. GFAP IHC demonstrates the diffuse astrogliosis associated with the neurodegenerative lesions. DAB immunoperoxidase labeling with hematoxylin counterstain. (c) Medulla oblongata from a 20-week-old NSG male. The neurodegenerative changes are associated with scattered CD45-positive mononuclear cells. DAB immunoperoxidase labeling with hematoxylin counterstain. (d) Pons from a 16-week-old NOG-EXL female. The neurodegenerative changes are associated with prominent overexpression of CD34, which highlights the bilateral symmetrical distribution of the affected regions. DAB immunoperoxidase labeling with hematoxylin counterstain. (e) and (f) Pons from a 32-week-old NOG female. Intralesional CD34 overexpression partially overlaps with glial markers including AIF-1 (arrows) and GFAP (asterisk) (multiplex immunofluorescence with AIF-1 [green], CD34 [red], GFAP [white], and DAPI counterstain [blue]).

IHC analysis for AIF-1 and GFAP confirmed that the neurodegenerative lesions were accompanied by microgliosis and astrogliosis (Fig. 2a-b and Supplemental Figures S2 and S3). Reactive microglial cells were often clustered around individual vacuoles (Fig. 2a). For comparison, constitutive levels of AIF-1 and GFAP immunoreactivity in the pons of normal NSG and C57BL/6 mice are shown in Supplemental Figure S2.

Scattered mononuclear cells expressing murine CD45 LCA were frequently observed within the reactive glial component (Fig. 2c), suggesting a neuroinflammatory response with the recruitment of peripheral monocytes/macrophages. In the CNS samples obtained from unaffected animals, including C57BL/6 control mice, CD45-expressing cells were extremely rare and usually identified within the vessels as circulating leukocytes.

While CD34 immunoreactivity was restricted to the vascular endothelium in the unaffected areas of the CNS (including the samples from the C57BL/6 controls) (Supplemental Figure S2), diffuse neuroparenchymal overexpression of CD34 was consistently observed in association with the neurodegenerative lesions (Fig. 2d and Supplemental Figures S2 and S3). In this context, CD34 appeared to be a sensitive and specific marker allowing a clear identification of minimally affected regions with subtle changes including small, scattered vacuoles and mild gliosis. CD34 IHC also highlighted the bilateral symmetrical distribution of the affected regions throughout the pons and medulla (Fig. 2d and Supplemental Figure S2). Multiplex IF confirmed partial colocalization between CD34 and glial markers including GFAP and AIF-1 (Fig. 2e and 2f). However, most of the signal involved focally extensive regions of the neuropil without any overlap with the glial markers (Fig. 2e, f).

As most of the mice included in the study were either treated with CAR T-cells or featured a humanized immune system, the potential contribution of human inflammatory/immune cells was also investigated via IHC using an antibody specific for the human CD45 LCA. Inflammatory/immune cells of human origin were not identified in any of the tested lesions ruling out the implication of a chimeric component in the development and/or progression of the neurodegenerative changes.

**Figure 3.**
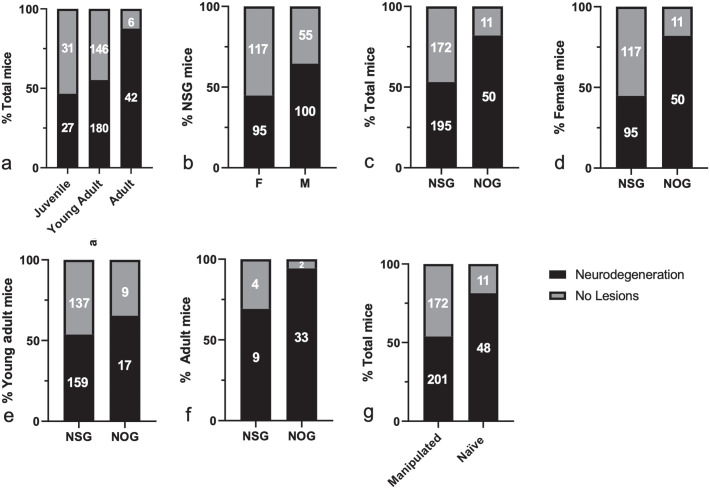
Frequency of spontaneous neurodegeneration in NSG, NOG, and NXG mice evaluated in this study. (a) Comparison of lesion frequency across different age categories (chi-square test for trend, *P* < .0001). (b) Comparison of lesion frequency in male and female NSG mice (Fisher’s exact test, *P* = .0002). (c) Comparison of lesion frequency in NSG and NOG mice (Fisher’s exact test, *P* < .0001). (d) Comparison of lesion frequency in NSG and NOG female mice (Fisher’s exact test, *P* < .0001). (e) Comparison of lesion frequency in NSG and NOG young adult mice (Fisher’s exact test, *P* = .3066). (f) Comparison of lesion frequency in NSG and NOG adult mice (Fisher’s exact test, *P* = .0385). (g) Comparison of lesion frequency in naïve and experimentally manipulated mice (Fisher’s exact test, *P* < .0001).

**Figure 4.**
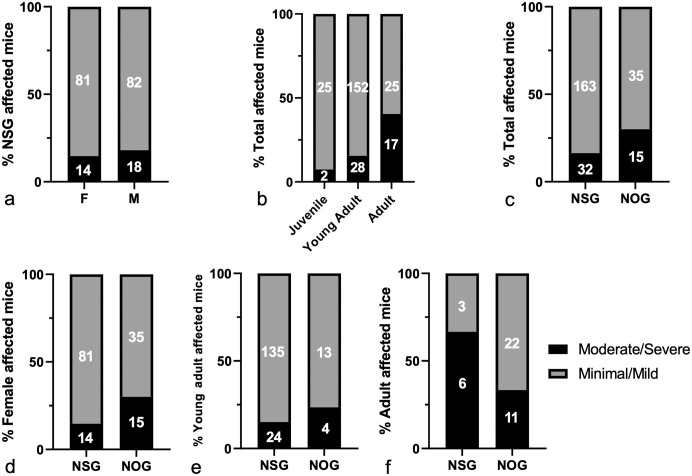
Severity of spontaneous neurodegeneration in NSG, NOG, and NXG mice evaluated in this study. (a) Comparison of lesion severity in male and female NSG mice (Fisher’s exact test, *P* = .5673). (b) Comparison of lesion severity across different age categories (chi-square test for trend, *P* = .0002). (c) Comparison of lesion severity in NSG and NOG mice (Fisher’s exact test, *P* = .0426). (d) Comparison of lesion severity in NSG and NOG female mice (Fisher’s exact test, *P* = .0477). (e) Comparison of lesion severity in NSG and NOG young adult mice (Fisher’s exact test, *P* = .4816). (f) Comparison of lesion severity in NSG and NOG adult mice (Fisher’s exact test, *P* = .1242).

Analysis of the frequency of neurodegenerative changes across different age groups revealed a statistically significant trend toward increasing morbidity in older mice (Fig. 3a). As neuropathological data concerning NOG or NXG males have not been included in this study, a comparative assessment of lesion frequency and severity between sexes was only performed in the NSG group. In this context, NSG males were more frequently affected than NSG females, but no difference in lesion severity was observed between sexes (Figs. 3b and 4a). Statistical analysis also indicated that lesions were significantly more common in NOG mice than in NSG mice (Fig. 3c). As expected, this difference between the 2 strains was even more prominent after excluding NSG males (Fig. 3d). However, the statistical significance was partially lost upon stratification based on age categories (Fig. 3e–f) implying that the variable frequency is influenced by the substantial age difference between the NSG and the NOG populations (Supplemental Figure S4a). Naïve/control animals were more frequently affected when compared with animals used for either humanization, tumor transplantation, or CAR T-cell treatment (Fig. 3g). This statistical outcome could not be linked to a substantial age difference between the 2 groups (Supplemental Figure S4b). Likewise, males (which were significantly more affected than females) were not overrepresented in the naïve group (Supplemental Figure S4c).

When assessing the severity of the neurodegenerative changes across different age categories, a significant trend was observed with older animals featuring more severe lesions (Fig. 4b). Overall, NOG mice were more severely affected than NSG mice (Fig. 4c). This difference between the 2 strains remained statistically significant after excluding NSG males (Fig. 4d). However, the significance was lost upon stratification based on age categories (Fig. 4e, f), implying that the variable severity was influenced by the substantial age disparity between the NSG and the NOG cohorts (Supplemental Figure S4a).

None of the animals included in this study showed neurological signs. Clinicopathological evidence of primary conditions (eg, infections or metabolic diseases) potentially associated with secondary neurodegenerative lesions was not observed. The review of the health monitoring reports from the mouse facilities where the studies were conducted between the 2013–2022 period did not identify any neurotropic infections including mouse cytomegalovirus (murine herpesvirus 1), murine polyomavirus, mouse parvovirus, lymphocytic choriomeningitis virus, lactate dehydrogenase-elevating virus, mouse hepatitis virus, and mouse encephalomyelitis virus .

## Discussion

This study contributes a comprehensive assessment of the frequency and histopathological characteristics of distinct neurodegenerative changes naturally occurring in NSG, NOG, and NXG mice. Importantly, this work incorporates neuropathological data collected in 10 years from different studies conducted in multiple facilities across Europe and the United States.

Our findings indicate that the neurodegenerative lesions can be found in juvenile animals as early as 6 weeks of age. Interestingly, both frequency and severity of the neurodegeneration appear to increase with age. This contrasts with prior descriptions of analogous “spongiotic” changes in the brainstem and spinal cord of NOG mice where the lesions were evident at 7 weeks of age, but they gradually disappeared in older animals between 26 and 52 weeks of age.^14^ Similarly, a previous study failed to report comparable CNS lesions in a large cohort of old NSG breeders (median age 52 weeks), suggesting that neurodegeneration might spontaneously resolve with age.^34^ Unfortunately, the limited age range of our study population prevented us from measuring the occurrence neurodegeneration in mice older than 37 weeks. For this reason, the age-related trend emerging from our statistical analysis requires further corroboration through the neuropathological assessment of older NSG, NOG, and NXG mice.

Our statistical analysis demonstrates that frequency, but not severity, of neurodegeneration is significantly increased in males compared with females. The comparison between sexes was possible only in the NSG population as no NOG or NXG males were included in the study. Notably, prior studies in NSG and NOG mice have revealed an analogous male predisposition for the development of similar neurodegenerative changes.^14,22^ Emphasizing the critical role of sex-related factors in determining the development and progression of CNS disorders, it has been recently documented that specific variations in microglial activation between sexes are responsible for the different susceptibility of male and female mice to experimental neurodegenerative conditions.^15,26,27^ Although this aspect is beyond the scope of our study and it has not been investigated, we speculate that sex-related changes in microglial activation might also contribute to the significant difference in lesion frequency between male and female NSG mice.

Overall, our findings suggest that NOG mice are more frequently and severely affected than NSG mice. The reasons behind this variation between the 2 strains are unclear and this finding, even if statistically significant, must be interpreted with caution given the substantial size difference and age range disparity existing between the NSG and NOG populations considered in this study. Nevertheless, previous investigations comparing these same strains have identified an analogous tendency with increased NOG predisposition for the development of similar spontaneous neurodegenerative changes.^22^

We observed that naïve/control animals were more frequently affected than experimentally manipulated animals. Even if difficult to reconcile, this evidence reinforces the notion of the spontaneous nature of the neurodegenerative changes described in our study. In addition, caution should be applied when interpreting this statistical outcome as the results may suffer again from the considerable size variation between groups.

While NXG mice were not included in the statistical analysis due to the extremely small number of mice available, importantly, we demonstrated that the same neurodegenerative lesions are also represented in this relatively new and still largely uncharacterized mouse line.

The definition of spontaneous lesions affecting commonly used laboratory mouse strains is critical for an accurate interpretation of pathological endpoints as it enables the discrimination among experimentally induced changes, background/incidental findings, and artifacts.^28,34,39,42^ This is particularly important in the case of the neuropathological changes described in our work as subtle lesions with minimal vacuolation can be easily misinterpreted as an artifact commonly seen in the histological preparations of the mouse CNS.^37^ Therefore, knowledge of the full spectrum of expected neuropathological changes is crucial to inform the need for more sensitive and specific approaches ruling out potential artifacts.^35^ In this context, we have been able to confirm the development of neurodegenerative changes via the identification of gliosis, neuronal degeneration/death, and immune/inflammatory cell infiltrate.

CD34 overexpression was one of the key features of the neuroparenchymal reaction associated with the CNS lesions described in our cohorts of NSG, NOG, and NXG mice. CD34 was shown to be a very sensitive and specific marker to identify subtle changes consisting of small, scattered vacuoles and minimal gliosis. The exact cell/tissue distribution of CD34 overexpression is uncertain. Focal colocalization between CD34 and AIF-1 or GFAP have been observed using multiplex IF. Nevertheless, CD34 signal also extended into the neuropil of the affected region without any overlap with the glial markers. CD34 is known to be ubiquitously expressed by vascular endothelial cells.^19^ Other cell types including endothelial progenitor cells and HSC are also known to be CD34-positive.^19,23,36,43^ CD34 overexpression by reactive microglia has been reported in the context of neurodegenerative conditions such as experimental amyotrophic lateral sclerosis in rats^16^ and traumatic brain injury in mice.^17^ However, evidence of CD34 expression in other CNS cell populations is scarce without a definitive identification of the cell origin.^19,21^

While our study does not address the specific cause(s) of neurodegeneration, we speculate on the possibility that its pathogenesis might be linked to an undetected viral infection. None of the typical neurovirulent pathogens were found to be circulating in the mouse facilities during the study period. However, the nature and distribution of the neurodegenerative lesions described in our study are reminiscent of the changes caused by mouse encephalomyelitis virus infection in immunocompromised mice.^32,33^ Retrovirus-induced spongiform neurodegeneration in mice also shares analogous pathological features with the lesions we described in NSG, NOG, and NXG mice.^3,4,18^ Importantly, it is widely recognized that severely immunocompromised mouse strains, such as the NSG, NOG, and NXG, can develop infections with previously unidentified viruses or microbes that are considered nonpathogenic in immunocompetent mice.^6,7,31^ In this context, much like the discovery of the novel mouse kidney parvovirus in NSG mice,^31^ metagenomic approaches should be undertaken to rule out the implication of previously unidentified viruses in the pathogenesis of the neuropathological changes reported in our study.

The possibility that the neurodegenerative phenotype observed in NSG, NOG, and NXG mice might result from the epistatic interaction between mutant alleles (ie, *Prkdc* and *Il2rg*), and/or NOD background represents another plausible hypothesis.^9,30^ Neither the individual *Prkdc* and *Il2rg* mutations nor the NOD background have been previously linked to the development of spontaneous neurodegeneration.^5,10,20,24,28,30^ Yet, neurodegeneration is invariably present when these genetic elements are combined in the NSG, NOG, or NXG mice suggesting that undetermined epistatic relationships might drive the development of the reported phenotype.

In conclusion, our work demonstrates the high prevalence of spontaneous early-onset neurodegeneration among NSG, NOG, and NXG mice from different mouse facilities across Europe and the United States. The specific cause(s) for this phenotype is(are) currently unclear, and further studies are necessary to determine the hypothesized involvement of unidentified pathogens as well as the role of epistatic interactions between mutations and allelic variations that are similarly represented in these mouse strains.

## Supplemental Material

sj-pdf-2-vet-10.1177_03009858231151403 – Supplemental material for Spontaneous early-onset neurodegeneration in the brainstem and spinal cord of NSG, NOG, and NXG mice Click here for additional data file. Supplemental material, sj-pdf-2-vet-10.1177_03009858231151403 for Spontaneous early-onset neurodegeneration in the brainstem and spinal cord of NSG, NOG, and NXG mice by Giovanni Finesso, Elinor Willis, James Carmine Tarrant, Matthew Lanza, Justin Sprengers, Jillian Verrelle, Esha Banerjee, Els Hermans, Charles-Antoine Assenmacher and Enrico Radaelli in Veterinary Pathology

sj-xlsx-1-vet-10.1177_03009858231151403 – Supplemental material for Spontaneous early-onset neurodegeneration in the brainstem and spinal cord of NSG, NOG, and NXG mice Click here for additional data file. Supplemental material, sj-xlsx-1-vet-10.1177_03009858231151403 for Spontaneous early-onset neurodegeneration in the brainstem and spinal cord of NSG, NOG, and NXG mice by Giovanni Finesso, Elinor Willis, James Carmine Tarrant, Matthew Lanza, Justin Sprengers, Jillian Verrelle, Esha Banerjee, Els Hermans, Charles-Antoine Assenmacher and Enrico Radaelli in Veterinary Pathology
