# Supplementary material for: Spontaneous early-onset neurodegeneration in the brainstem and spinal cord of NSG, NOG, and NXG mice
Source: Vet Pathol. 2023 Feb 2;60(3):374–83. doi: 10.1177/03009858231151403 (PMC10150263; doi:10.1177/03009858231151403)
Supplement: sj-pdf-2-vet-10.1177_03009858231151403 – Supplemental material for Spontaneous early-onset neurodegeneration in the brainstem and spinal cord of NSG, NOG, and NXG mice [file sj-pdf-2-vet-10.1177_03009858231151403.pdf]

Supplemental Materials

Spontaneous early onset neurodegeneration in the brainstem and spinal cord of NSG, NOG, and NXG mice

Giovanni Finesso, Elinor Willis, James Carmine Tarrant, Matthew Lanza, Justin Sprengers, Jilliane Verrelle, Esha Banerjee, Els Hermans, Charles-Antoine Assenmacher, Enrico Radaelli

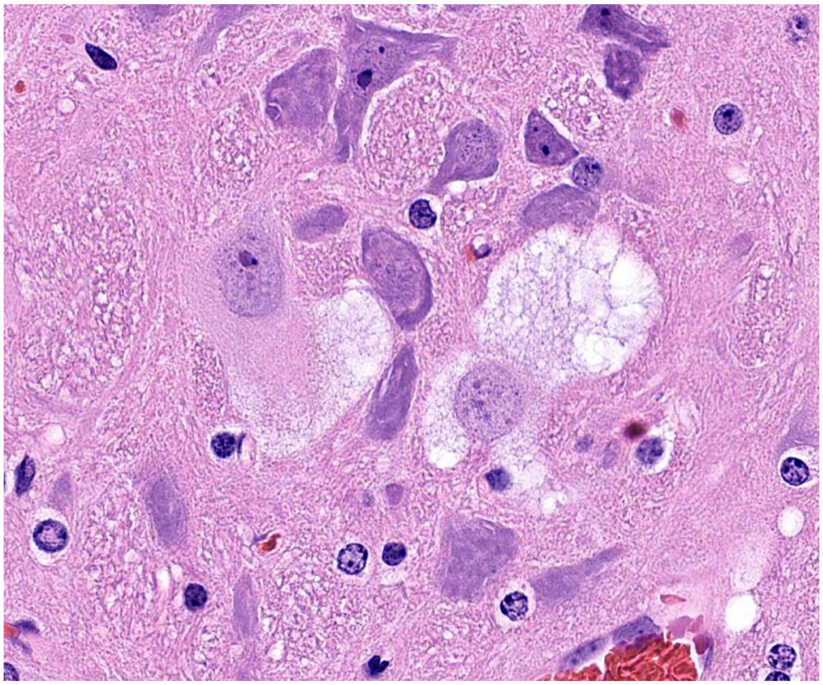

Supplemental Figure S1. Histopathologic features of spontaneous neurodegeneration in NSG, NOG, and NXG mice. Pons from a 25-week-old NOG female. High power magnification of degenerating neurons characterized by enlarged and distorted perikaryon with foamy cytoplasm (HE).

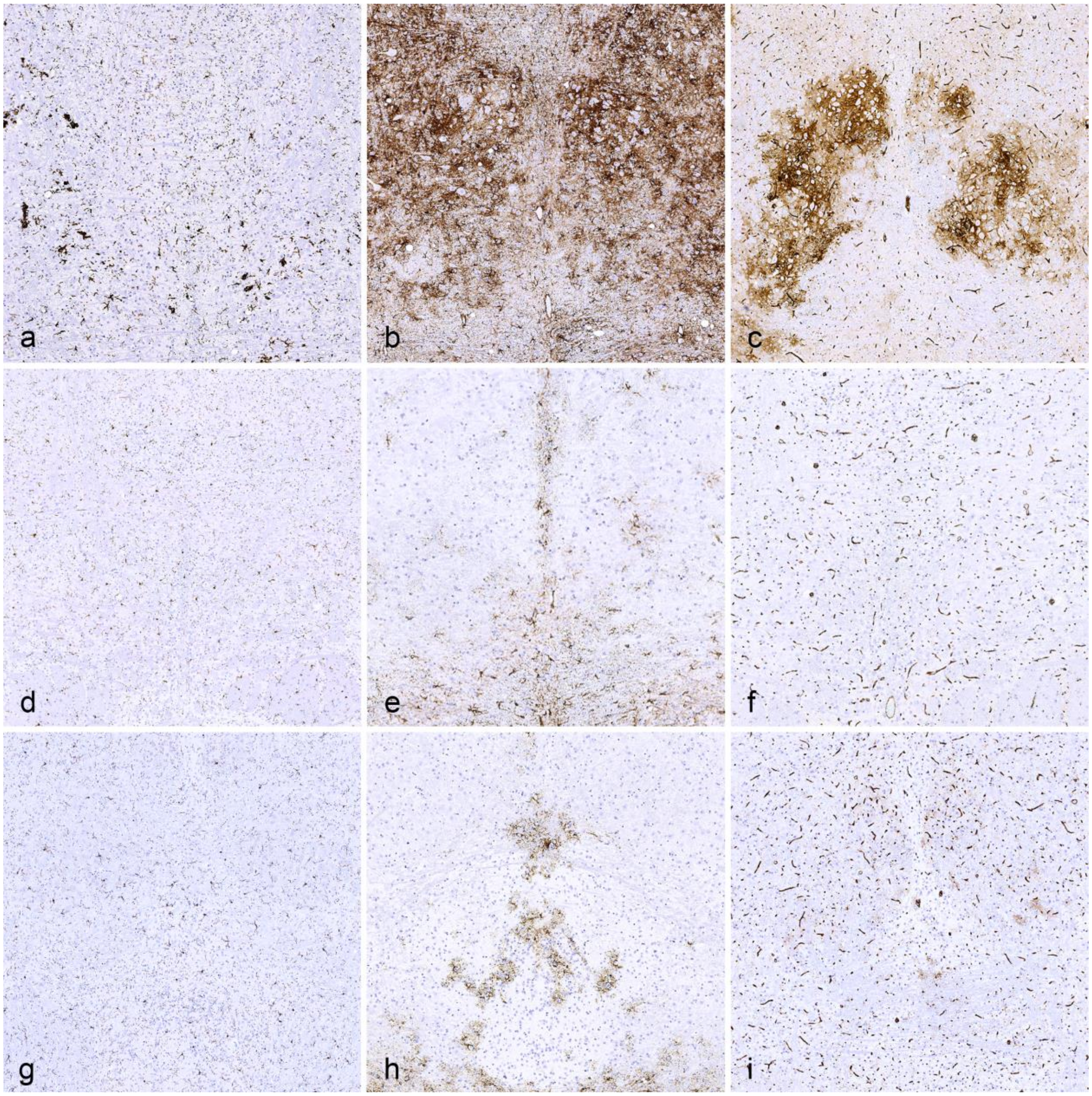

Supplemental Figure S2. Immunohistochemical features of spontaneous neurodegeneration in NSG, NOG, and NXG mice. Pons from an affected 25-week-old NSG male showing a) microgliosis with increased AIF-1 expression, b) astrogliosis with increased GFAP expression, and c) CD34 overexpression. Pons from a normal 30-week-old C57BL/6J male showing constitutive level of d) AIF-1, e) GFAP, and f) CD34 expression (DAB immunoperoxidase staining with hematoxylin counterstain). Pons from an unaffected 20-week-old NSG male showing level of g) AIF-1, h) GFAP, and i) CD34 expression comparable to the normal C57BL/6J mouse.

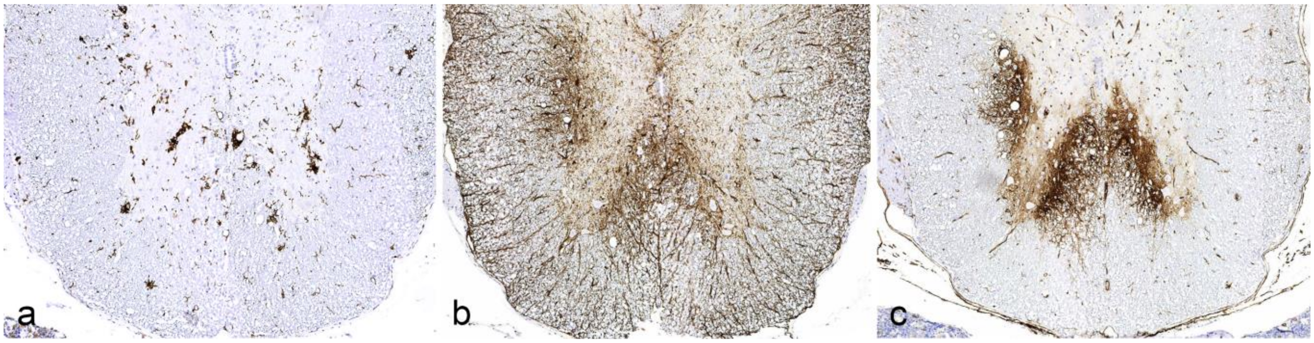

Supplemental Figure S3. Immunohistochemical features of spontaneous neurodegeneration in NSG, NOG, and NXG mice. Thoracic spinal cord from an 11-week-old NSG female (same sample depicted in Fig. 1b) showing that the affected regions along the gray/white matter junction of ventral horns and intermediate gray are characterized by a) microgliosis with increased AIF-1 expression, b) astrogliosis with increased GFAP expression, and c) CD34 overexpression (DAB immunoperoxidase staining with hematoxylin counterstain).

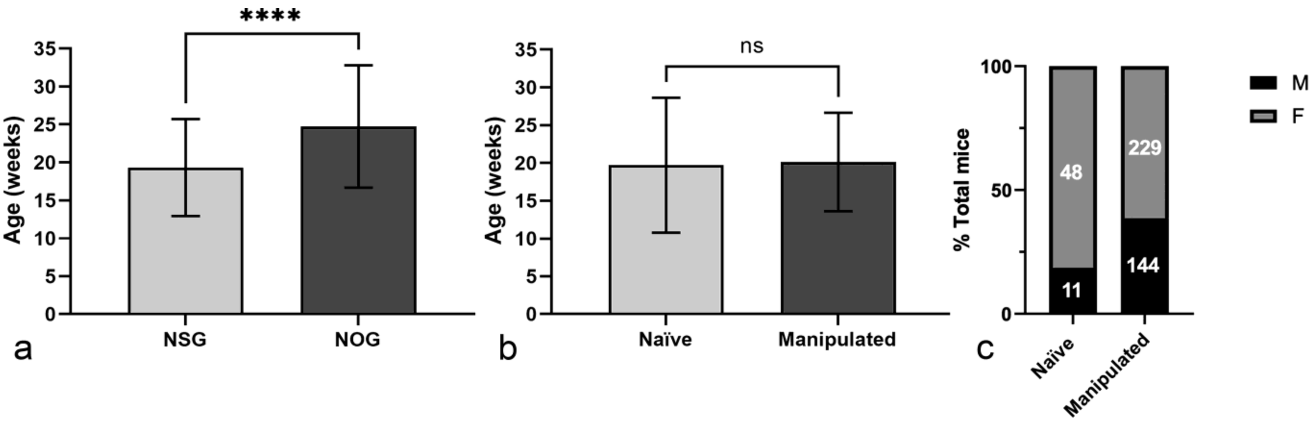

Supplemental Figure S4. a) Comparison of the average age between NSG and NOG mice (two-tailed Student's *t*-test, $p<0.0001$). b) Comparison of the average age between naïve and experimentally manipulated mice (two-tailed Student's t-test, $p=0.6742$). c) Comparison of the male/female ratio in naïve and experimentally manipulated mice Fisher's exact test, $p=0.0032$).

Supplemental Table S1. Overview of the mouse cohorts included in the study summarizing demographic data as well as frequency and severity of the neurodegenerative changes based on the different sex groups, age categories, experimental use, and anatomical distribution
Provided in separate Excel file.

Supplemental Table S2. Details concerning primary antibodies and procedures used for chromogenic immunohistochemistry and multiplex immunofluorescence

Antigen/ Target	Primary antibody	Source	Antigen retrieval	Working dilution	Incubation time	Positive (+) and negative (-) controls	Staining platform and detection system	Number of cases
AIF-1	Rb mAb #019-19741	Wako	Sodium citrate based pH 6.0 solution (#AR9961), 20 minutes, 98 °C	1:1200 (IHC), 1:3000 (IF)	45 minutes RT	(+) Brain from an adult C57BL/6J mouse<br>(-) Incubation with an irrelevant isotype-matched Rb mAb	Leica BOND RXm, Bond Polymer Refine Detection Kit (#DS9800) (IHC), Opal 3-Plex Detection Kit (NEL820001KT) (IF)	(a) 12 NSG, 3 NOG, 1 NXG<br>(u) 4 NSG, 6 C57BL/6
GFAP	Rb mAb #80788	Cell Signaling Technology	EDTA based pH 9.0 solution (#AR9640), 20 minutes, 98 °C	1:800 (IHC), 1:3000 (IF)	45 minutes RT	(+) Brain from an adult C57BL/6J mouse<br>(-) Incubation with an irrelevant isotype-matched Rb mAb	Leica BOND RXm, Bond Polymer Refine Detection Kit (#DS9800) (IHC), Opal 3-Plex Detection Kit (NEL820001KT) (IF)	(a) 5 NSG, 3 NOG, 1 NXG<br>(u) 4 NSG, 3 C57BL/6
CD34	Rb mAb #ab81289	Abcam	Sodium citrate based pH 6.0 solution (#AR9961), 20 minutes, 98 °C	1:200 (IHC), 1:600 (IF)	45 minutes RT	(+) Brain from an adult C57BL/6J mouse<br>(-) Incubation with an irrelevant isotype-matched Rb mAb	Leica BOND RXm, Bond Polymer Refine Detection Kit (#DS9800) (IHC), Opal 3-Plex Detection Kit (NEL820001KT) (IF)	(a) 5 NSG, 3 NOG, 1 NXG<br>(u) 4 NSG, 3 C57BL/6
CD45 LCA (mouse specific)	Rt mAb #553076	BD Biosciences	EDTA based pH 9.0 solution (#AR9961), 20 minutes, 98 °C	1:300 (IHC)	45 minutes RT	(+) Lymphoid tissues from an adult Crl:CD1(ICR) mouse<br>(-) Incubation with an irrelevant isotype-matched Rt mAb	Leica BOND RXm, Bond Polymer Refine Detection Kit (#DS9800) (IHC)	(a) 7 NSG, 3 NOG<br>(u) 4 NSG, 3 C57BL/6
CD45 LCA (human specific)	Rb mAb. #13917	Cell Signaling Technology	EDTA based pH 9.0 solution (#AR9961), 20 minutes, 98 °C	1:300 (IHC)	45 minutes RT	(+) Human tonsil<br>(-) Incubation with an irrelevant isotype-matched Rb mAb	Leica BOND RXm, Bond Polymer Refine Detection Kit (#DS9800) (IHC)	(a) 7 NSG, 3 NOG<br>(u) 4 NSG, 3 C57BL/6

Abbreviations used in the table: Rb-Rabbit, Rt-Rat, mAb-Monoclonal Antibody, HIER-heat induced epitope retrieval, min-minutes, RT-room temperature, IHC-chromogenic immunohistochemistry, IF-multiplex immunofluorescence, (a) affected, (u) unaffected.
